# Systemic-to-pulmonary collateral flow in patients with palliated univentricular heart physiology: measurement using cardiovascular magnetic resonance 4D velocity acquisition

**DOI:** 10.1186/1532-429X-14-25

**Published:** 2012-04-27

**Authors:** Israel Valverde, Sarah Nordmeyer, Sergio Uribe, Gerald Greil, Felix Berger, Titus Kuehne, Philipp Beerbaum

**Affiliations:** 1 Division of Imaging Sciences and Biomedical Engineering, King’s College London. NIHR Biomedical Research Centre at Guy’s & St Thomas’ NHS Foundation Trust, 4th Floor Lambeth Wing, St. Thomas Hospital, SE1 7EH, London, UK; 2 Department of Congenital Heart Diseases, Evelina Children’s Hospital, Guy’s & St Thomas’ NHS Foundation Trust, Westminster Bridge Road, London, UK; 3 Department of Congenital Heart Disease and Paediatric Cardiology, Deutsches Herzzentrum Berlin, Unit of Cardiovascular Imaging, Berlin, Germany; 4 Radiology Department, Pontificia Universidad Católica de Chile, Santiago de Chile, Chile; 5 Department of Pediatric Cardiology, Charité Universitaetsmedizin Berlin, Berlin, Germany

## Abstract

**Background:**

Systemic-to-pulmonary collateral flow (SPCF) may constitute a risk factor for increased morbidity and mortality in patients with single-ventricle physiology (SV). However, clinical research is limited by the complexity of multi-vessel two-dimensional (2D) cardiovascular magnetic resonance (CMR) flow measurements. We sought to validate four-dimensional (4D) velocity acquisition sequence for concise quantification of SPCF and flow distribution in patients with SV.

**Methods:**

29 patients with SV physiology prospectively underwent CMR (1.5 T) (n = 14 bidirectional cavopulmonary connection [BCPC], age 2.9 ± 1.3 years; and n = 15 Fontan, 14.4 ± 5.9 years) and 20 healthy volunteers (age, 28.7 ± 13.1 years) served as controls. A single whole-heart 4D velocity acquisition and five 2D flow acquisitions were performed in the aorta, superior/inferior caval veins, right/left pulmonary arteries to serve as gold-standard. The five 2D velocity acquisition measurements were compared with 4D velocity acquisition for validation of individual vessel flow quantification and time efficiency. The SPCF was calculated by evaluating the disparity between systemic (aortic minus caval vein flows) and pulmonary flows (arterial and venour return). The pulmonary right to left and the systemic lower to upper body flow distribution were also calculated.

**Results:**

The comparison between 4D velocity and 2D flow acquisitions showed good Bland-Altman agreement for all individual vessels (mean bias, 0.05±0.24 l/min/m^2^), calculated SPCF (−0.02±0.18 l/min/m^2^) and significantly shorter 4D velocity acquisition-time (12:34 min/17:28 min,p < 0.01). 4D velocity acquisition in patients versus controls revealed (1) good agreement between systemic versus pulmonary estimator for SPFC; (2) significant SPCF in patients (BCPC 0.79±0.45 l/min/m^2^; Fontan 0.62±0.82 l/min/m^2^) and not in controls (0.01 + 0.16 l/min/m^2^), (3) inverse relation of right/left pulmonary artery perfusion and right/left SPCF (Pearson = −0.47,p = 0.01) and (4) upper to lower body flow distribution trend related to the weight (r = 0.742, p < 0.001) similar to the controls.

**Conclusions:**

4D velocity acquisition is reliable, operator-independent and more time-efficient than 2D flow acquisition to quantify SPCF. There is considerable SPCF in BCPC and Fontan patients. SPCF was more pronounced towards the respective lung with less pulmonary arterial flow suggesting more collateral flow where less anterograde branch pulmonary artery perfusion.

## Background

**Figure 1:**
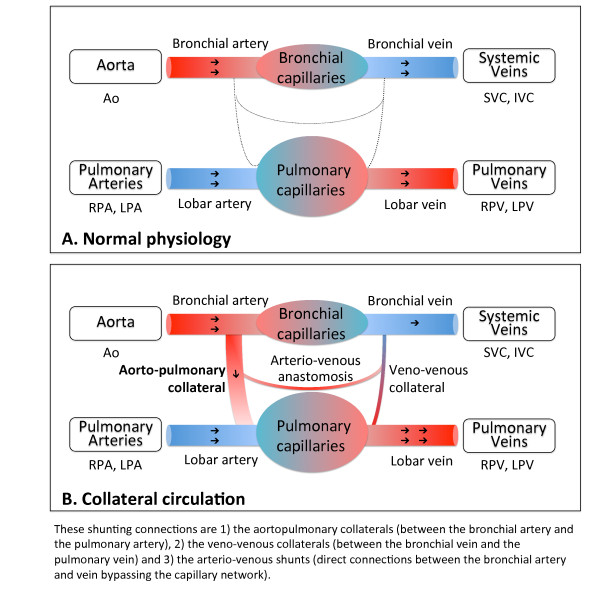
**Scheme of systemic and pulmonary circulation. (A)** Normal physiology: The virtual network connections are present but are not permeable. **(B)** Collateral circulation: There is a shunt network between the systemic and the pulmonary circulation. These shunting connections are 1) the aortopulmonary collaterals (between the bronchial artery and the pulmonary artery), 2) the veno-venous collaterals (between the bronchial vein and the pulmonary vein) and 3) the arterio-venous shunts (direct connections between the bronchial artery and vein bypassing the capillary network). *Adapted from Heimburg P*[4]*, copyright notice 2011, with permission from BMJ Publishing Group Ltd.*

Systemic-to-pulmonary collateral flow (SPCF, Figure 1) often develops in patients with univentricular heart physiology after bidirectional cavopulmonary connection (BCPC) or Fontan-type palliation although little is known about their true prevalence [1]. Hemodynamically, SPCF may result in competitive pulmonary perfusion and power loss in the Fontan pathway by transferring kinetic energy to the distal pulmonary vasculature, and in volume loading of the systemic single-ventricle [2]. The relevance of SPCF in terms of morbidity and mortality of patients with univentricular heart physiology remains controversial due to lack of reproducible quantitative noninvasive methods to assess SPCF. Recently, Whitehead and colleagues introduced a new method to non-invasively quantify SPCF using two-dimensional phase-contrast (2D flow) cardiac magnetic resonance (CMR) velocity mapping in single-ventricle patients after superior BCPC [2]. Two different estimators of SPCF were proposed, namely, the difference between aortic and caval flow (systemic estimator), and the difference between pulmonary venous and pulmonary arterial flow (pulmonary estimator); and close agreement was observed for both approaches. This allows for internal validation of SPCF quantification, which is highly important, as no other gold-standard method exists [2]. However, this technique (as well as similar approaches [3]) is complex as multiple 2D flow measurements are required to determine SPCF (both caval veins, ascending/descending aorta, branch pulmonary arteries, pulmonary veins). Hence, although non-invasive and quantitative, this technique is lengthy and highly dependent on operator skills, which makes it cumbersome for larger-scale prospective clinical research needed to further, elucidate the clinical role of SPCF after staged repair of single-ventricle physiology.

In this context, we propose the use of whole-heart four-dimensional (4D) velocity acquisition phase-contrast CMR flow to quantify the SPCF contributing to pulmonary perfusion. The 4D velocity acquisition scan can be planned as a simple box covering the whole mediastinal cardiovascular system. The sequence has been already validated for healthy adults [5], but not for patients with single-ventricle physiology. Therefore, the purpose of this two-centre prospective study is firstly to validate the use of 4D velocity acquisition for non-invasive quantification of SPCF against 2D flow measurement [3,6] in patients after BCPC or Fontan-type palliation; and secondly, from the validated 4D velocity acquisition data, to compare the systemic and pulmonary estimator for SPCF between patients and controls. We hypothesized that [1] there would be more SCPF in BCPC than Fontan, [2] that anterograde versus collateral pulmonary perfusion of either lung might be inversely related, and [3] that increased SPCF would correlate to increased end-diastolic ventricular volumes [2].

## Methods

### Study population

The institutional review boards of both institutions approved all protocols and written and signed consent for research and publishing purposes was obtained from each patient or their legal guardians.

This prospective two-centre study included 29 successive patients with univentricular heart physiology who were referred for routine CMR investigation at either Evelina Children’s Hospital, Guy’s & St. Thomas’ Hospitals in London, United Kingdom (12 BCPC, 8 Fontan) or at the German Heart Institute in Berlin, Germany (2 BCPC, 7 Fontan) between March 2010 and February 2011.

The BCPC group (n = 14) mean age was 2.9 ± 13 years, with a female/male ratio of 8/6. The Fontan group (n = 15) mean age was 14.4 ± 5.9 years and the female/male ratio was 4/11. No patient had previous diagnosis or suspicion of relevant SPCF. Exclusion criteria were: Arrhythmia, inlet or outlet valvular incompetence, residual flow across surgical shunts, residual anterograde flow into the pulmonary artery. The demographic data are summarized in Table 1. Additionally, 20 controls (mean age 28.7 ± 13.1 years, 9 females / 11 males) underwent 4D velocity acquisition scanning to evaluate the presence of SPCF (n = 13 controls at Guy’s & St. Thomas’ Hospital, n = 7 at the German Heart Institute, Berlin).

**Table 1:** Summary of the patients’ demographic data, primary diagnosis and type of palliated surgery

	**BCPC**	**Fontan**	***p value***
Age at CMR (years)	2.9 ± 1.3	14.4 ± 5.9	0.01*
Weight (kg)	12.5 ± 3.1	46.2 ± 22	0.01*
BSA (m^2^)	0.5 ± 0.1	1.4 ± 0.4	0.01*
Females (%)	8 (57 %)	4 (27 %)	>0.05
Age at BCPC (years)	0.6 ± 0.2	1.1 ± 0.8	0.01*
Time between BCPC – CMR (years)	2.3 ± 1.3	11.4 ± 3.1	0.01*
Age at Fontan (years)	-	5.7 ± 6.5	-
Time between BCPC-Fontan (years)	-	2.9 ± 1.3	-
Time between Fontan – CMR (years)	-	8.6 ± 4.1	-
**Primary cardiac diagnosis**			
Double inlet left ventricle	1	4	n/a
Tricuspid atresia	1	3	n/a
PA – IVS	2	-	n/a
HLHS	10	5	n/a
Unbalanced AVSD	1	2	n/a
Straddling AV valve	-	1	n/a
**Staged palliated surgery**			
Hemi-Fontan	11	-	n/a
BCPC	3	-	n/a
Classic Fontan (Atriopulmonary connection)	-	4	n/a
Intracardiac Lateral tunnel	-	2	n/a
Extracardiac Conduit	-	9	n/a

AV, atrioventricular valve; AVSD, atrioventricular septal defect; BCPC, bidirectional cavopulmonary connection; BSA, body surface area; CMR, cardiovascular magnetic resonance; HLHS, hypoplastic left heart syndrome; PA - IVS, pulmonary atresia and intact ventricular septum; TCPC, total cavopulmonary connection; n/a, not applicable; *, p < 0.05.

### CMR studies

All CMR scans were performed on a whole-body 1.5 T Achieva MR scanners (Philips Medical Systems, Best, The Netherlands) with either a 5-channel or 32-channel cardiac surface coil. Patients younger than 10 years were examined under general anaesthesia or conscious sedation. All patients underwent clinical CMR investigations according to a uniform study protocol to investigate the cardiovascular anatomy, ventricular function (multi-slice steady-state free precession) and patency of the BCPC or Fontan circuits. Additionally, hemodynamic quantification of SPCF was investigated by using phase-contrast CMR 2D and 4D velocity acquisition as detailed below. The controls underwent 4D velocity acquisition scanning for validations purposes but no 2D flow acquisitions as 4D velocity acquisition versus 2D flow validation has been published previously [5].

#### Two-dimensional phase-contrast flow

Standardized localizer imaging planes were first acquired to plan 2D flow acquisitions across five targeted vessels: superior vena cava (SVC), inferior vena cava (IVC), right pulmonary artery (RPA), left pulmonary artery (LPA) and ascending aorta (AO). Care was taken to align the plane perpendicular to flow and to obtain slice positions that were inferior to the vena azygos insertion into the SVC, and midway between pulmonary bifurcation and distal branching for both pulmonary arteries. Free-breathing 2D phase-contrast sequences were then obtained in the five targeted vessels using the CMR parameters described in Table 2.

**Table 2:** CMR parameters for 2D and 4D velocity acquisition scans

	**2D velocity acquisition**	**4D velocity acquisition**
Field of view (mm)	150 x 300	200 x 300
Acquired voxel size (mm)	2.3 x 2.3 x 7	2.4 x 2.5 x 2.5
Reconstructed voxel size (mm)	1.2 x 1.2 x 7	1.5 x 1.5 x 2.3
Number of slices	1	25-40
Cardiac gating	Retrospective	Retrospective
Respiratory motion	Non-gated	Non-gated
	Free breathing	Free breathing
NSA	2	1
TR(ms)/TE(ms)	5/3	3.2 /1.9
Flip angle (°)	10	5
SENSE	No	2
Reconstructed cardiac phases	35-40	22-25
VEC (cm/s)	60-100 (venous vessels) 200–400 (arterial vessels)	150-400

2D, two-dimensional; 4D, four-dimensional; CMR, cardiovascular magnetic resonance; NSA, number of signal averages; SENSE, sensitivity encoding for fast CMR; TR, repetition time; TE, echo time; VEC, velocity encoding.

#### Four-dimensional velocity acquisition

A free-breathing non-respiratory-gated 4D velocity acquisition sequence covering the whole heart and great vessels within the mediastinum was acquired using the CMR parameters detailed in Table 2. The maximal velocity encoded values (VENC) were predefined based on the maximal velocity measured in the analyzed vessels by previous echocardiography. The same VENC was set in the three spatial directions. For 4D and 2D phase-contrast flow scans, the time for both data acquisition and scan planning was measured. Repeated 2D flow acquisitions due to plane misalignment or velocity aliasing were also included in the total time.

### Flow data post-processing

2D flow analysis was performed in an Extended MR Workspace station (Version 2.5.3.1, Philips Healthcare, Best, The Netherlands). The region of interest in each targeted vessel was manually traced in every cardiac phase to obtain the average flux along one cardiac cycle, indexed to body surface area (BSA, l/min/m^2^).

**Figure 2:**
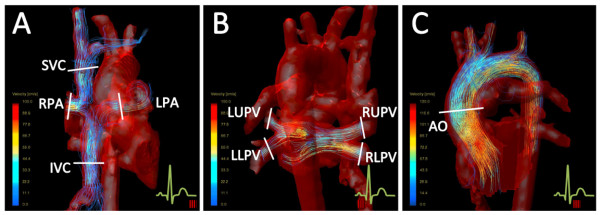
**4D velocity acquisition plane location for flow investigation. (A)** Anterior view of the Fontan circuit and aortic arch with visualized pathlines within the IVC, LPA, RPA and SVC. **(B)** Posterior view of the Fontan circuit, aortic arch and pulmonary veins with visualized pathlines within the pulmonary veins. **(C)** Sagittal view of the aortic arch with visualized pathlines. *AO, aorta; IVC, inferior vena cava; LLPV, left lower pulmonary veins; LPA, left pulmonary artery; LUPV, left upper pulmonary veins; RLPV, right lower pulmonary veins; RPA, right pulmonary artery; RUPV, right upper pulmonary veins.*

The 4D velocity acquisition data was analyzed using the software ‘GTFlow’ (Release 1.5.4, Gyrotools, Zurich, Switzerland). The 4D velocity acquisition data was reformatted along the five targeted vessel using the geometry imported from the 2D imaging planes (Figure 2). Thereafter, the region of interest was then traced manually in the same way as for 2D flow acquisition. Additionally, flux was also obtained in the individual right pulmonary veins (RPV) and left pulmonary veins (LPV) by manually reformatting the 4D velocity acquisition data into a plane perpendicular to each vessel.

All 2D and 4D velocity acquisitions were assessed by two independent observers with over three years of experience in CMR.

### Statistical analysis and calculations

Continuous variables are presented as mean ± standard deviation (SD). Statistical analysis was performed using SPSS software (version 17; SPSS, Chicago, Ill). A p-value less than 0.05 was considered to indicate statistically significant differences. Demographic data differences between patient groups were evaluated by Student t-test and Chi-square test.

#### Validation of 4D versus 2D velocity acquisition

The agreement between 2D flow acquisition and 4D velocity acquisition for the five individual vessels flow in patients with univentricular heart physiology was evaluated by Bland-Altman plot analysis and their correlation assessed by Pearson correlation analysis. Intra- and interobserver variance for repeated 2D and 4D velocity acquisition vessel measurements was evaluated by intraclass correlation coefficient (ICC). Time difference between 2D flow and 4D velocity acquisitions was evaluated by paired t-test.

#### Evaluation of SPCF

The SPCF can be calculated by evaluating the systemic flow estimator [AO-(SVC-IVC)] or the pulmonary flow estimator [(RPV + LPV)-(RPA-LPA)] disparity (see Table 3). The agreement between 2D and 4D velocity acquisition for SPCF calculation using the systemic flow method was evaluated by Bland-Altman plot analysis and their correlation by the Pearson correlation analysis, as was the internal 4D velocity acquisition validation for the systemic versus pulmonary flow estimator of SPCF. Evaluation of quantitative SPCF for BCPC/Fontan/controls was assessed by paired t-test. Multiple regression analysis was used to evaluate significant correlation of SPCF with independent variables (age at BCPC, time since the BCPC operation, age at Fontan, time since Fontan operation, ventricular end-diastolic volume and pulmonary [Qp] to systemic [Qs] flow ratio).

**Table 3:** Calculated parameters blood flow parameters

	**Derived equations**
**Systemic blood flow (Q**_**S**_**)**	
Traditional (Systemic arterial supply)	AO
New (Systemic venous return)	SVC + IVC
**Pulmonary blood flow (Q**_**P**_**)**	
Traditional (Pulmonary arterial supply)	RPA + LPA
New (Pulmonary venous return)	RPV + LPV
**Systemic-to-pulmonary collateral flow (SPCF)**	
Systemic flow estimator	(AO) – (SVC + IVC)
Pulmonary flow estimator	(RPV + LPV) - (RPA + LPA)

AO, aorta; IVC, inferior vena cava; LPA, left pulmonary artery; LPV, left pulmonary veins; RPA, right pulmonary artery; RPV, right pulmonary veins; SPCF, systemic-to-pulmonary collateral flow; SVC, superior vena cava.

#### Pulmonary right to left flow distribution

The distribution of the blood flow for BCPC/Fontan/controls between the right and left lung was evaluated by 4D velocity acquisition in terms of pulmonary arterial flow (RPA + LPA) and venous return (RPV + LPV) (Table 3) and evaluated by paired t-test. The Pearson test was performed to evaluate the SPCF and pulmonary artery flow correlation.

#### Systemic lower to upper body flow distribution

**Figure 3:**
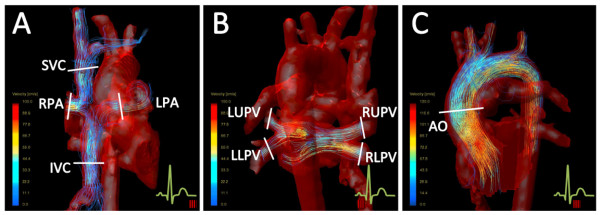
**Systemic lower to upper body flow ratio in patients and controls.** Inferior vena cava to total systemic venous flow ratio [IVC:(IVC+SVC)] to show the lower to upper body flow relationship changes with body weight. The patient’s data (circle) and trend-line (R2=0.406) shows similar distribution to the control data (squares).

The percentage of IVC (%) related to the total systemic venous return [IVC/(IVC + SVC)*100] was also evaluated for patients with univentricular heart physiology and controls (Figure 3). A multiple regression analysis was used to evaluate the correlation of IVC-percentage with independent variables (weight, height and BSA).

## Results

### Baseline characteristics

Patient’s characteristics are summarized in Table 1. BCPC and Fontan patients were significantly different in terms of age at the investigation, weight and body surface area (BSA) (p < 0.001). Age at BCPC surgery was significantly lower in the BCPC group than in the Fontan group. The mean age of the control group was 28.7±13.1 years, mean weight 66±19 kg and mean BSA 1.7±0.4 m^2^. All 49 CMR investigations were completed successfully. There were no statistically significant differences in the female to male ratio between BCPC, Fontan and Control groups (p = 0.245). Twenty 2D flow sequences were repeated due to plane malalignment or velocity aliasing. No 4D sequence had to be repeated. The average time to satisfactorily obtain the five individual 2D flow scans (17:28±04:24 min) was significantly longer than the single 4D velocity acquisition sequence (12:34±03:42 min, p < 0.01). The mean indexed end-diastolic and end-systolic ventricular volumes were 85.8 ± 24.2 ml/m^2^ and 36.4 ± 19.8 ml/m^2^ for BCPC and 91.7 ± 21 ml/m^2^ and 41 ± 15.7 ml/m^2^ for Fontan patients respectively. The ejection fraction was 61.8 ± 8 % for the BCPC and 56.4 ± 10.3 % for Fontan patients. In 15 patients we found some degree of atrioventricular valve incompetence (mild to moderate).

### Validation of 4D velocity acquisition versus 2D flow measurements in patients

**Figure 4:**
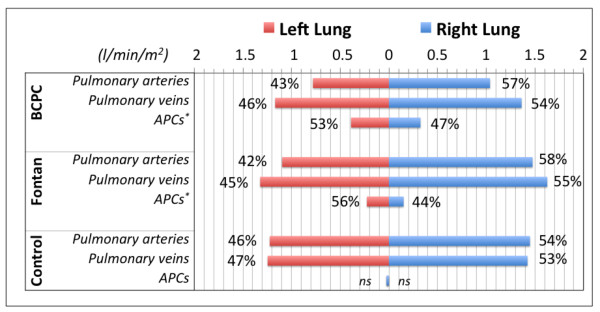
**2D and 4D velocity acquisition comparison charts in patients with univentricular heart physiology. A.** Bland-Altman agreement graph. **B.** Correlation scatter-plot. 2D, two-dimensional; 4D, four-dimensional; AO, aorta; IVC, inferior vena cava; LPA, left pulmonary artery; RPA, right pulmonary artery; SVC, superior vena cava.

In all patients, 4D velocity acquisition and the 2D flows were comparable for all investigated vessels (Bland-Altman mean difference 0.05±0.24 l/min/m^2^) as shown in Table 4 and Figure 4A. This was also reflected by the excellent Pearson coefficient (Table 4) and correlation trend-line (R^2^ = 0.88, Figure 4B). Intra- and interobserver variability for all the individual vessels was excellent for 2D velocity flow (ICC > 0.97, 95 % confidence interval 0.96-0.99) and also for 4D velocity acquisition (ICC > 0.95, 95 % confidence interval 0.91-0.97). The calculated systemic-to-pulmonary collateral flow (SPCF) by systemic estimator (AO)–(SVC + IVC) [2] in patients with univentricular heart physiology showed good agreement between 2D velocity acquisition and 4D velocity acquisition (Bland-Altman analysis, mean difference −0.02±0.18 l/min/m^2^) with good correlation (Pearson correlation coefficient 0.73, p < 0.05, Table 4). The 4D velocity acquisition internal validation SPCF calculation by systemic versus pulmonary estimator (Table 3) showed good agreement with some scatter (mean bias 0.01±0.78 l/min/m^2^). We chose the pulmonary estimator method as it allowed depicting the venous return for both lungs, and it was used subsequently for the 4D velocity acquisition-based sub-analyses as detailed below.

**Table 4:** Patients with univentricular heart physiology: Mean values and agreement of 2D and 4D velocity acquisition measurements

	**BCPC**		**Fontan**		**Univentricular heart physiology**	
	*2D velocity acquisition*	*4D velocity acquisition*	*2D velocity acquisition*	*4D velocity acquisition*	*Bland-Altman*	*Pearson*
					*difference*	*Correlation*
					*2D - 4D velocity acquisition*	*2D - 4D velocity acquisition*
**SVC**	1.77±0.61	1.62±0.61	0.93±0.34	0.92±0.35	0.07 ± 0.04	0.96 *
**IVC**	1.79±1.01	1.74±1.05	1.80±0.63	1.64±0.55	0.11 ± 0.01	0.93 *
**RPA**	1.06±0.37	1.01±0.36	1.43±0.51	1.47±0.59	0.01 ± 0.05	0.91*
**LPA**	0.79±0.37	0.77±0.44	1.12±0.28	1.10±0.33	0.02 ± 0.05	0.94*
**AO**	3.61±1.21	3.57±1.16	3.08±0.70	3.04±0.56	0.04 ± 0.05	0.94*
**SPCF**	0.59±0.52	0.79±0.45	0.41±0.46	0.62±0.82	−0.02 ± 0.18	0.73*
**Qp:Qs**	(0.43±0.23):1	(0.42±0.23):1	(0.82±0.29):1	(0.84±0.26):1	(0.02 ± 0.18):1	0.82*

Values expressed as l/min/m^2^. *statistically significant correlation; 2D, two-dimensional; 4D, four-dimensional, AO, aorta; BCPC, bilateral cavopulmonary connection; CMR, cardiovascular magnetic resonance; IVC, inferior vena cava; LPA, left pulmonary artery; Qp, pulmonary blood flow; Qs, systemic blood flow; RPA, right pulmonary artery; SPCF, systemic-to-pulmonary collateral flow (SPCF = AO-SVC-IVC); SVC, superior vena cava.

### 4D velocity acquisition for SPCF: patients versus controls

Table 5 summarizes the flow data in controls. There was no significant SPCF in the control group (−0.01±0.16 l/min/m^2^, p > 0.05). However, there was significant SPCF using the pulmonary estimator (SPCF, Figure 4) in patients with BCPC (0.79±0.45 l/min/m^2^, p < 0.05) and Fontan (0.56±0.81 l/min/m^2^, p < 0.05). For BCPC patients, SPCF represented 25.8±20.2 % of total Qp (pulmonary venous return), and 17.8±15.4 % of total Qs (aortic outflow). For Fontan patients, SPCF represented 19.7±26.4 % of total Qp (pulmonary venous return) and 21.0±26.8 % of total Qs (aortic outflow).

**Table 5:** Controls: demographics and 4D CMR flow data

**Demographics**	
Controls (n)	20
Age at CMR (years)	28.7±13.1
Weight (kg)	65.9±19.2
BSA (m^2^)	1.7±0.4
Females (%)	9 (41 %)
**4D velocity acquisition** (*l/min/m*^*2*^*)*	
SVC	0.91±0.14
IVC	1.8±0.43
RPA	1.48 ± 0.28
LPA	1.26 ± 0.25
RPV	1.45 ± 0.29
LPV	1.28 ± 0.25
AO	2.74±0.45
SPCF	−0.01±0.16

4D, four-dimensional; AO, aorta; BSA, body surface area; CMR, cardiovascular magnetic resonance; IVC, inferior vena cava; LPA, left pulmonary artery; LPV, left pulmonary veins; RPA, right pulmonary artery; RPV, right pulmonary veins; SVC, superior vena cava; SPCF, systemic-to-pulmonary collateral flow (SPCF = RPV + LPV-RPA-LPA).

In our patient cohort, SPCF magnitude was not associated with ventricular end-diastolic or end-systolic volume, ventricular ejection fraction, age at (or time since) BCPC/Fontan operation, respectively.

#### 4D velocity acquisition: pulmonary right to left flow distribution

**Figure 5:**
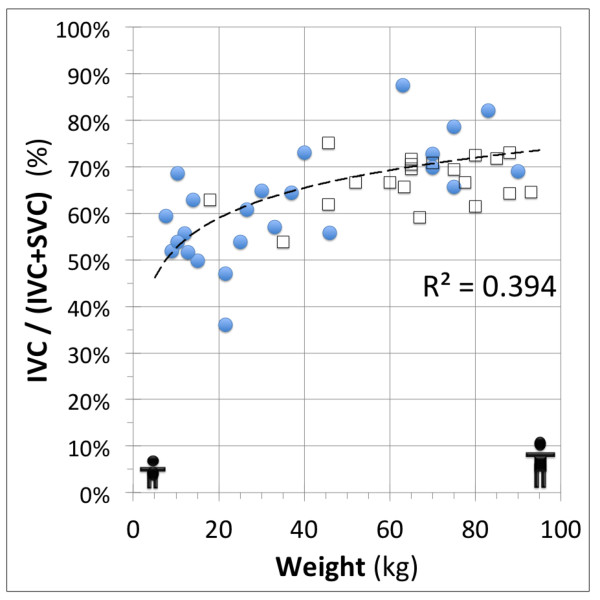
**Pulmonary right to left lung blood flow distribution calculated by 4D velocity acquisition evaluated in terms of arterial blood supply (pulmonary arteries) and venous return (pulmonary veins).** The flow difference between the pulmonary veins and the pulmonary arteries is represented by the systemic aortopulmonary collaterals (APC). *=Statistically significant difference.

For BCPC and Fontan patients, there was a preferential flow via the pulmonary arteries to the right lung, as seen in the control group (p < 0.01, Figure 5). Preferential SPCF however was towards the left lung (p < 0.05), with inverse correlation of pulmonary artery inflow and SPCF to the respective lung (Pearson = −0.48, p < 0.05).

#### 4D velocity acquisition: systemic venous flow distribution (IVC/SVC)

The IVC to total systemic venous return percentage [(IVC/(IVC + SVC)*100] changed from 50 % in younger patients up to 75 % in larger patients (Figure 3). A multiple regression analysis including age, weight, height and BSA revealed that the weight is the best independent variable to predict the IVC percentage ratio in patients with univentricular heart physiology (r = 0.742, p < 0.001). This trend was also seen in the controls (Figure 3).

## Discussion

### 4D versus 2D velocity acquisition for SPCF quantification in single-ventricle palliation

In this first study using 4D velocity acquisition to assess quantitative pulmonary perfusion after univentricular heart palliation study, we have shown that 4D velocity acquisition-based SPCF determination is simple, more time-effective and accurate when compared with 2D velocity acquisition. Previous validation of 4D velocity acquisition against the gold-standard of 2D velocity acquisition was mainly performed in adult volunteers [5,7] with only a small number of pediatric patients with miscellaneous congenital heart diseases included [5]. 4D velocity acquisition technique had a good observer reproducibility and agreement with 2D velocity acquisition being the current gold-standard method for vessel flow quantification [8]. 4D velocity acquisition allowed for straight-forward internal validation of calculated SPCF by either systemic flow or pulmonary flow estimator (see Table 3) [2]. In this clinical setting, 4D velocity acquisition has principle advantages over 2D velocity acquisition: [1] In a single and easy-to-plan scan, all vessels of interest are acquired; [2] investigation of unsuspected vascular connections not appreciated during the scanning procedure are eligible to quantitative evaluation during post-processing which is obviously not possible with 2D velocity acquisition; [3] free image-plane reformation during post-processing allows investigation at any vessel location avoiding stent artifacts or velocity aliasing. Hence, our data suggest superiority of 4D velocity acquisition over conventional multi-site 2D velocity acquisition to quantify SPCF in staged Fontan-type palliation and may replace 2D CMR flow in this important clinical setting.

### Clinical impact

In this context, due to its simplicity, the 4D velocity acquisition method may prove useful in prospective clinical research to elucidate the clinical importance of SPCF magnitude in the evolution of the failing Fontan circulation. Although the sample size was relatively small for each group, our study generated first data in this context using the 4D velocity acquisition approach, which relates to previous findings and provides new information.

1. We found no significant SPCF in n = 20 controls. Due to the variety of applied methods to quantify SPCF, it has previously been difficult to establish ‘normal’ SPCF values although some numbers were reported to be in the order of 7 % of the cardiac output [9].

2. There was significant SPCF in our two patient groups. In the BCPC group we measured 0.71±0.57 l/min/m^2^ which represented 25.8±20.2 % of total Qp (=pulmonary venous return) whilst in the Fontan group SPCF was slightly less with 0.56±0.81 l/min/m^2^ representing 19.7±26.4 % of total Qp. In other words, SPCF contributed around 18-21 % of the total systemic (aortic) flow. This amount of left-to-right shunting is considerable albeit not massive, and hence it was no surprise that we were unable to find any correlation of SPCF magnitude with single ventricle sizes or systolic function (i.e., end-diastolic/end-systolic volumes and ejection fraction) for either patient group. This is in contrast with findings published recently by Whitehead and colleagues who did observe such correlation [2] but available sample sizes from both studies (<20 subjects for each respective patient group) may be too small to allow meaningful conclusions in either direction in terms of relevance of SPCF for progressive ventricular dilatation and dysfunction in Fontan patients. This will require much larger numbers and a multicenter study design with consistent operator-independent flow quantification and central core-lab image reading facilities. We feel that the proposed validated 4D velocity acquisition technique may be useful for such an undertaking.

3. In our unselected group of BCPC and Fontan patients, the observed SPCF numbers were generally smaller than previously reported by using other methods for quantification. In the BCPC group for example, the SPCF was previously reported as mean of 1.75±0.46 l/min/m^2^ by a combined approach with nuclear imaging and catheterization [10] whilst Grosse-Wortmann et al. using CMR 2D velocity acquisition recently suggested a median 0.78 and 1.42 l/min/m^2^, depending on whether either the systemic estimator or the pulmonary estimator was used for calculation [3]. Whitehead et al. did not observe such discrepancy in their cohort of 17 BCPC patients and reported an average indexed SPCF of 0.5 to 2.8 l/min/m^2^ (=11 % to 53 % (mean, 37 %) of aortic flow, and 19 % to 77 % (mean, 54 %) of pulmonary venous return. In our study we observed in the Fontan patient cohort, our calculated SPCF (mean 0.56±0.81 l/min/m^2^) was comparable to that by Groose-W et al. [3] (median 0.82 l/min/m^2^). These discrepancies are likely due to methodological constrains as explained above, but also possibly due to selection bias with higher likelihood of inclusion of patients with known aorto-pulmonary collateral arteries when investigating SPCF quantification.

4. Our study corroborates the reported regression in the magnitude of SPCF from BCPC to Fontan stages [3]. In patients with BCPC, where the pulmonary blood flow is limited to nearly half of the venous return (SVC), the development of SPCF is greater than in Fontan patients (SPCF and Qp:Qs regression analysis, r = −0.47, p = 0.01).

5. Interestingly, we observed an inverse relation of anterograde pulmonary artery inflow and the magnitude of the SPCF to the respective lung (Pearson −0.48, p = 0.001). In accordance with previously reported data [6], we found preferential anterograde flow towards the right lung, which was slightly more pronounced in BCPC (57.1 % to RPA versus 42.9 % to LPA, p = 0.07) than in the Fontan patients (56.9 % versus 43.1 %, p = 0.01) and in the control group (54.9 % versus 45.1 %, p = 0.001). It is tempting to speculate that the SPCF develops predominantly towards lung territories with relatively reduced anterograde arterial perfusion. Although this would need confirmation in larger series with a wider range of disparate right/left lung arterial perfusion, it seems to underscore the clinical experience of more collateralization in more severely underperfused lungs. It has been suggested that a combination of elements [3] such as reduced blood flow to one region of the lungs [4], reduced pulsatility and velocity profiles [11], high transpulmonary gradient or systemic undersaturation [12] or humoral factors [13] might be involved, but this still remains unclear.

6. In terms of the increase in IVC fraction of total systemic venous return over time, this is the first study to include both BCPC and Fontan patients. Our data are consistent with previous studies in normal children [14] and Fontan patients [6], reflecting that changes in systemic blood flow distribution is barely affected by staged palliated surgery.

Finally, although not focus of the present study, we also would like to state the great potential of 4D velocity acquisition for visualization of blood flow patterns allowing to estimate kinetic energy distribution and qualifying to set up boundary conditions for computational fluid dynamic research [15]. The evaluation of particle traces in Fontan patients has been performed systematically in previous studies[16,17] and may help understanding the low mechanics and in-efficient hemodynamics which may contribute to the pathophysiology of the failing Fontan circulation (See Additional file 1: Video S1 and Additional file 2: Video S2). In future studies a combination of APC flow quantification and description of flow patterns in relation to clinical outcome in patients with univentricular hearts might be very promising.

### Limitations

It is known that 4D velocity acquisition can be subject to error from non-flow-related phase shifts due to eddy currents and concomitant gradient fields, limited temporal and spatial resolution and respiratory compensate motion [5,7,18]. Although more research is needed to quantify such effects, 4D velocity acquisition is a novel technique under continuous development and improvement (for example, time-efficient respiratory gating and novel undersampling strategies to improve acquisition speed), and hence we expect even higher levels of accuracy in future application [15]. For venous and Fontan pathway flows, the settings of velocity-encoding values (VENC) were higher for 4D velocity acquisition than in targeted 2D velocity acquisition scans which may have contributed to some of the observed scatter [18]. Due to the presence of atrioventricular valve regurgitation we could not include a comparison analysis between ventricular stroke volumes from multi-slice steady-state free precession with those obtained from 4D velocity encoded in the aorta.

The use of mechanical ventilation and intravenous Propofol for general anaesthesia in younger patients could have lead to altered flow through the pulmonary and systemic circulations. One could speculate that higher intrathoracic pressure leads to reduced passive venous return through the Fontan circulation, combined with reduced systemic pressure this might lead to a reduction in overall APC flow.

It is a known dilemma that the majority of magnetic resonance scanners are positioned supinely. Thus, the influence of gravity on Fontan flow cannot be studied accurately with MR imaging. However, previous studies have used Doppler imaging to assess the influence of gravity on Fontan flow. The work of Hsia et al. [19] indicates that gravity decreases net venous flow and increases retrograde venous flow in Fontan patients. Since in our study, Hemifontan and Fontan patients were studied in supine position, the amount of APC flow might be different compared to the physiologically more relevant upright position.

In our study we used a standardized protocol, in which 4D flow measurements were always performed after the 2D flow measurements. The time difference between both flow measurements was approximately 15 minutes, thus, we believe it is unlikely that a relevant bias was introduced, however, a systematic error of this approach cannot be fully excluded.

## Conclusions

We have shown that 4D velocity acquisition is a reliable and accurate technique, which is more time efficient than 2D velocity acquisition for quantitative analysis of systemic and pulmonary perfusion including SPCF after palliation of single-ventricle physiology. SPCF was found to be present in both BCPC and Fontan patients and approximates 20-26 % of pulmonary venous return, and 18-21 % of aortic output. There was no obvious association of SPCF with ventricular dilatation or systolic function. There was an inverse relation of branch pulmonary arterial flow and SPCF to the respective lung, suggesting that SPCF may develop predominantly where anterograde flow is reduced (or vice versa). The 4D velocity acquisition approach has great potential beyond these observations to add further valuable information about kinetic energy distribution and aid computer fluid dynamics modeling approaches to optimize Fontan pathway flow dynamics. Hence, 4D velocity acquisition method may be useful in prospective studies to investigate pulmonary flow mechanics including SPCF to evaluate their impact on outcome late after palliated univentricular heart physiology.

## Competing interests

The authors declare that they have no competing interests.

## Author’s contributions

IV and SN participated in the design of the study and the MR scanning of patients and volunteers (acquisition of the data). They performed all measurements and calculations and drafted the manuscript. SU had performed measurements in healthy volunteers, helped to analyse data and participated in revising the manuscript. GG participated in the design and coordination of the study in London and helped to acquire the data and revise the manuscript. FB participated in the design and coordination of the study in Berlin and revised the manuscript critically. TK participated in the design and coordination of the study in Berlin, helped to analyse and interpret the data and to draft the manuscript. PB initiated the design and coordination of the study, participated in the analysis and interpretation of the data and drafted the manuscript. All authors read and approved the final manuscript.

## Supplementary Material

Additional file 1 **Video S1.Particle trace in the Fontan circuit.** Particle trace released in the Fontan circulation in a patient with hypoplastic left heart syndrome. Visual examination of the particles provides insight about the flow dynamics. Note the whirl flow originated in the confluence between the superior and the inferior vena cava. Some particles from the inferior vena cava shunt to the right atrium through the fenestration. Click here for file

Additional file 2 **Video S2.Particle trace in the pulmonary veins and aorta.** Particle trace evaluation in the pulmonary veins and aorta in a patient with hypoplastic left heart syndrome. Note the blood flow entering into the ventricle and being pumped through the aorta. The laminar flow is well preserved in the ascending and transverse arch with no major areas of turbulent flow seen. Click here for file
